# Cytotoxic Effects of *Bifora testiculata* (L.) Spreng. Essential Oil and Its Main Component on Cancer Cell Lines

**DOI:** 10.3390/plants14152408

**Published:** 2025-08-04

**Authors:** Alessandro Vaglica, Antonella Porrello, Natale Badalamenti, Vincenzo Ilardi, Maurizio Bruno, Filippo Maggi, Massimo Bramucci, Luana Quassinti

**Affiliations:** 1 Department of Biological, Chemical and Pharmaceutical Sciences and Technologies (STEBICEF), Università degli Studi di Palermo, Viale delle Scienze, Ed. 17, 90128 Palermo, Italy; alessandro.vaglica@unipa.it (A.V.); antonella.porrello@unipa.it (A.P.); natale.badalamenti@unipa.it (N.B.); vincenzo.ilardi@unipa.it (V.I.); maurizio.bruno@unipa.it (M.B.); 2 National Biodiversity Future Center (NBFC), 90133 Palermo, Italy; 3 Chemistry Interdisciplinary Project (ChIP) Research Center, School of Pharmacy, University of Camerino, Via Madonna delle Carceri, 62032 Camerino, Italy; filippo.maggi@unicam.it; 4 School of Pharmacy, University of Camerino, Via Gentile III da Varano, 62032 Camerino, Italy; massimo.bramucci@unicam.it

**Keywords:** *Bifora testiculata* (L.) Spreng, Apiaceae, aldehydes, anticancer agent, oxidative stress, apoptosis

## Abstract

*Bifora testiculata* (L.) Spreng. (Apiaceae), an understudied species endemic to the Mediterranean and the only representative species of the genus *Bifora* in Sicily, was investigated for the first time for its essential oil (EO) chemical composition and cytotoxic properties. The EO was obtained via hydrodistillation and analyzed using GC-MS, revealing an aldehyde-rich profile (86.10%), dominated by *trans*-2-dodecenal (67.49%). Comparative analysis with previous studies on *B. testiculata* from Greece confirmed a similar aldehyde-rich profile, although minor compositional differences suggest potential chemotype variation. Given the biological relevance of *trans*-2-dodecenal and related aldehydes, further investigations into the cytotoxic properties of the EO of *B. testiculata* (**Bt**) and its main constituent against cancer cell lines were undertaken. Three human tumor cell lines (MDA-MB 231, A375, and CaCo2) and a human non-tumor cell line (HEK293) were subjected to viability tests using the MTT assay. The EO and *trans*-2-dodecenal exhibited remarkable cytotoxic activity against all cell lines, with IC_50_ values ranging between 7.93 and 14.41 µg/mL for **Bt** and between 1.88 and 5.29 µg/mL for *trans*-2-dodecenal. AO/BE fluorescent staining and Hoechst nuclear staining showed the presence of apoptotic bodies in the treated cells. *N*-acetyl-L-cysteine was able to invert the effects of **Bt** and *trans*-2-dodecenal on cell lines, suggesting ROS involvement in cytotoxic activity. The results demonstrated that the **Bt** cytotoxic activity was mainly due to the presence of *trans*-2-dodecenal.

## 1. Introduction

The genus *Bifora* Hoffm. is a member of the Apiaceae family and is classified under the tribe Coriandreae [1]. This widely distributed yet disjunct genus comprises three species: two species found across Eurasia, and one native to North America. *B. radians* M. Bieb. extends from Spain through southern and central Europe, reaching as far east as Iran. *B. testiculata* (L.) Spreng. has a broader and more southern range, spanning from Spain to Uzbekistan and extending to North Africa, the Levant, and Saudi Arabia. Meanwhile, *B. americana* (DC.) A. Gray is native to the southern United States, predominantly Texas and Arkansas, with reports of introduction in Alabama [2].

*B. radians* has been traditionally used as a culinary spice and folk remedy in parts of Turkey, where its aerial parts are known as *yabani kişniş otu* or *kokarot* and are used for their stomachic and carminative effects [3,4,5]. In contrast, in Jordan, *B. testiculata*, known used under the name كزبرة (*kazbira*), is used under infusion together with coriander for its sedative and stomach-soothing properties [6]. While these ethnobotanical uses highlight the relevance of *Bifora* species in traditional medicine, scientific interest has predominantly focused on *B. radians*, especially for its insecticidal activity against agricultural pests [7,8,9,10,11] and for its antimicrobial effects against *Escherichia coli*, *Pseudomonas aeruginosa*, *Bacillus subtilis*, *Staphylococcus aureus*, and *Candida albicans* [12,13]. Moreover, its EO, rich in aliphatic aldehydes, has demonstrated antibacterial activity against *Paenibacillus larvae* and insecticidal properties against the aphid *Lipaphis pseudobrassicae* [14].

**Figure 1:**
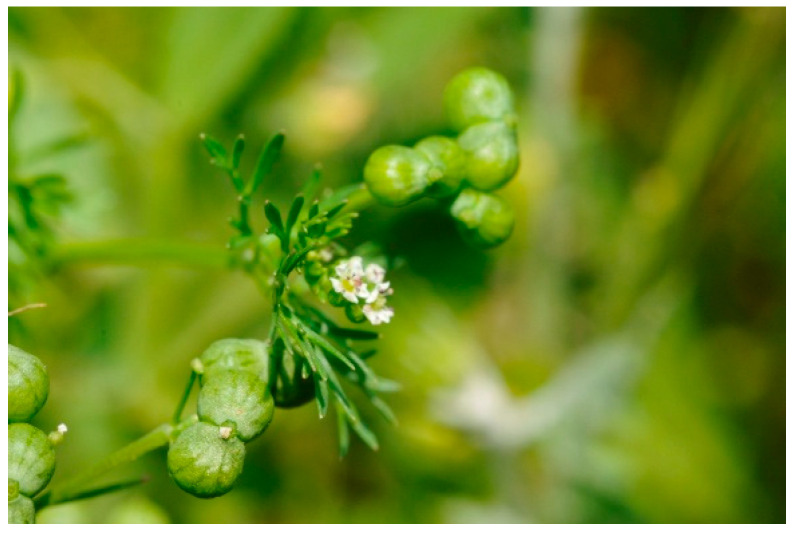
Morphological aspects of *Bifora testiculata* collected in Sicily (Serre di Villalba, Caltanissetta, Italy), showing aerial parts used for EO extraction. Photo by Prof. Vincenzo Ilardi.

*B. testiculata* [syn.: *Anidrum testiculatum* (L.) Kuntze; *Atrema testiculatum* (L.) Miq.; *Coriandrum testiculare* Salisb.; *Coriandrum testiculatum* L.; *Corion testiculatum* (L.) Hoffmanns. & Link; *Sium testiculatum* (L.) Vest] (Figure 1) is an herbaceous annual plant that grows to a height of 20–40 cm. It is glabrous and typically exhibits freely branched stems. The leaves are one- to two-pinnate, oblong in outline, with upper leaves having linear lobes that may be flat, entire, or lobed. The rays can extend up to 10 mm, while the bracts are absent or reduced to a single bract with 2–3 subulate bracteoles. The flowers have nearly equal-sized petals, and the fruit is rugose, measuring approximately 2.5–3.5 mm long and 4.5–7 mm wide, with a short beak. The style, typically less than 0.2 mm long, is as long as the stylopodium [15].

Despite extensive research on *B. radians*, *B. testiculata* remains largely unexplored, particularly regarding its biological and pharmacological properties. Given the increasing interest in plant-derived compounds for cancer treatment, the potential cytotoxic effects of *B. testiculata* EO (**Bt**) and its major components warrant investigation. Essential oils (EOs) from various Apiaceae species have demonstrated significant bioactivities, including cytotoxic effects on cancer cells [16]. The EO of plants rich in aldehydes has exhibited anti-cancer activity [17]. Cinnamon EO is endowed with a wide range of pharmacological functions, including antioxidant, antimicrobial, and anti-cancer activities. The main compound, cinnamaldehyde, has been characterized as an anti-cancer drug [18]. Citronellal, the main compound in the EO of *Cymbopogon* species, has been found to inhibit the growth of hypopharyngeal carcinoma cells, which indicates its potential as a drug for this type of cancer [19].

This study aimed to bridge this knowledge gap by evaluating the cytotoxic properties of **Bt** and providing new insights into its therapeutic applications. Therefore, **Bt** and its main components were tested against human breast adenocarcinoma, human malignant melanoma, and human colon adenocarcinoma cell lines, three of the common solid tumor cancers today.

## 2. Results and Discussion

### 2.1. Chemical Composition of **Bt**

Hydro-distilled **Bt**, obtained from aerial parts collected in Sicily, Italy, was pale yellow. Overall, twenty-six different compounds were identified (Table 1).

**Figure 2:**
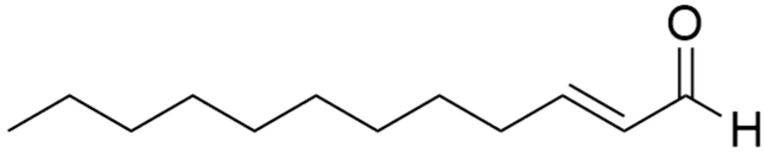
Chemical formula of *trans*-2-dodecenal.

**Bt** was particularly rich in aliphatic aldehydes, which accounted for 86.10% of its total composition. The predominant compound, *trans*-2-dodecenal (Figure 2), was the most abundant, representing 67.49% of the total EO. Other aldehydes present in noteworthy percentages included dodecanal (4.50%), *trans*-4-undecenal (3.38%), *cis*-2-decenal (3.25%), and *cis*-2-dodecenal (2.91%). Additionally, a moderate percentage of aliphatic acids (5.38%) was detected, with *trans*-2-dodecenoic acid (4.55%) being the most abundant. In contrast, monoterpenes, hydrocarbons, and other metabolites were found only in traces.

Table 2 presents the major compounds (>3%) identified in the EOs of all *Bifora* taxa reported in the literature.

To date, only one study has reported the chemical composition of **Bt** based on plants collected in Greece [20]. Similar to our findings, that study showed a high percentage of aldehydes, with *trans*-2-dodecenal (56.3%) and dodecanal (4.0%) found in comparable proportions to those observed in **Bt**. However, Evergetis et al. [20] reported significant percentages of *trans*-2-tridecenal (16.1%) and heptadecanal (14.0%), which were virtually absent in the **Bt**. This suggests possible chemotype variations between different geographical populations of the species.

Studies on the EOs of other *Bifora* species, such as *B. radians*, have shown the presence of aldehydes as the principal constituents [18,19,20]. However, in these cases, the major constituents were *trans*-2-tridecenal (47.2–66.4%) and *trans*-2-tetradecenal (14.6–24.6%).

Despite the differences in specific aldehyde compositions, the consistently high aldehyde content observed in both *B. testiculata* and *B. radians* suggests that aldehydes may serve as taxonomic markers for the genus *Bifora*.

**Table 2:** Main constituents (>3%) of EOs from *Bifora* species reported in the literature.

Taxa	Origin, Parts	Compounds	Ref.
*B. radians* Bieb.	Turkey, ap	*trans*-2-tridecenal (47.2), *trans*-2-tetradecenal (23.4), tridecanal (5.9), *trans*-2-dodecenal (5.8)	[21]
*B. radians* Bieb.	Cultivated, ap	*trans*-2-tridecenal (66.4), *trans*-2-tetradecenal (14.6), *trans*-2-dodecenal (10.7)	[22]
*B. radians* Bieb.	Turkey, ap	*trans*-2-tridecenal (52.9), *trans*-2-tetradecenal (24.6)	[23]
*B. testiculata* (L.) Spreng.	Greece, ap	*trans*-2-dodecenal (56.3), *trans*-2-tridecenal (16.1), heptadecanal (14.0), dodecanal (4.0)	[20]

ap = aerial parts.

### 2.2. Effects of **Bt** on Tumor Cell Viability

**Figure 3:**
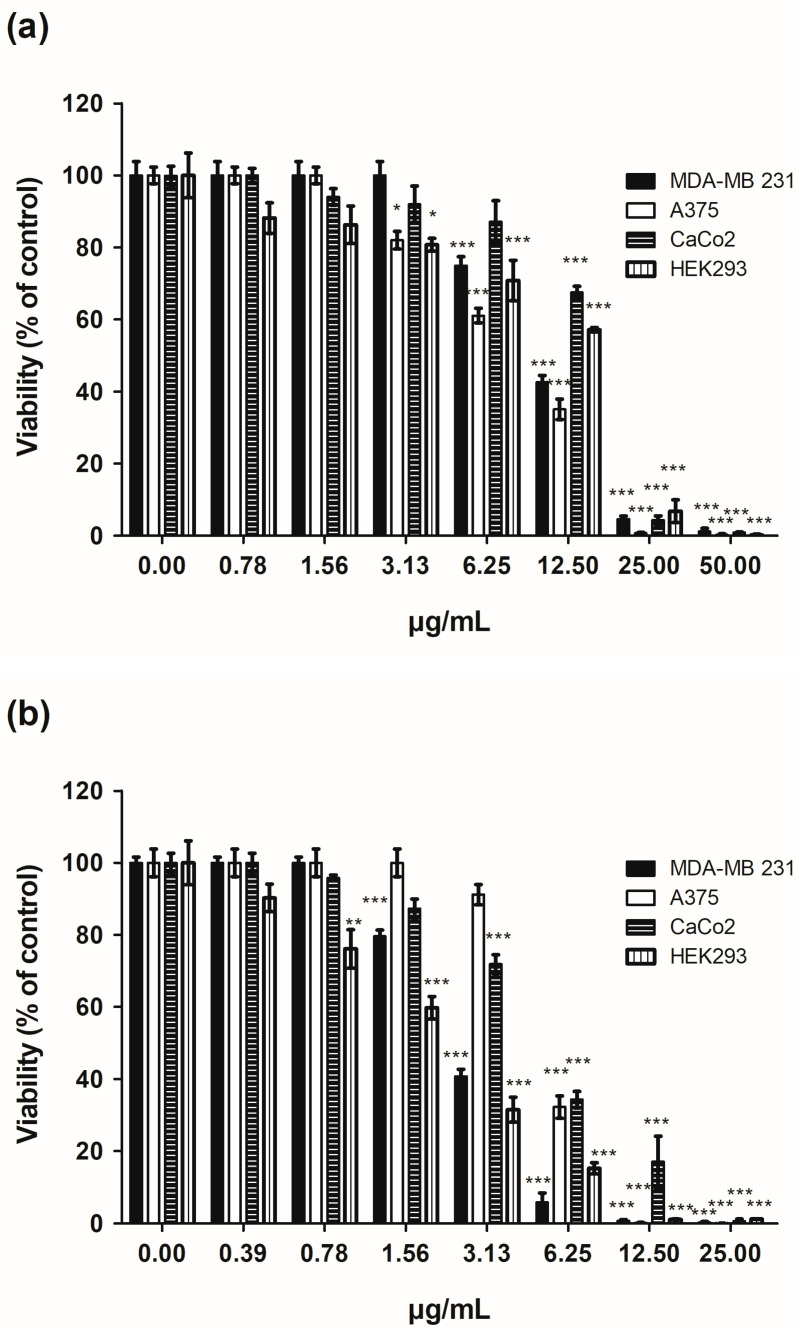
Dose-dependent cytotoxic effects of **Bt** (**a**) and *trans*-2-dodecenal (**b**) on human tumor (MDA-MB 231, A375, and CaCo2) and non-tumor cell lines (HEK293) after 72 h of treatment. Cell viability was measured using the MTT assay. Data are presented as mean ± SE of three independent experiments. * *p* < 0.05, ** *p* < 0.01, *** *p* < 0.001 (vs vehicle).

To the best of our knowledge, no data are available on the cytotoxic activity of **Bt**. In this study, the cytotoxic activity of **Bt** and its main component, *trans*-2-dodecenal, was tested against a panel of three human tumor cell lines and one non-tumor cell line. Human breast cancer MDA-MB 231, human malignant melanoma A375, human colon adenocarcinoma CaCo2 cell lines, and non-tumor human embryonic kidney cell line HEK293 were used to determine the effects of **Bt** and its principal constituents on the viability of cells. All cell lines were exposed to treatments in the presence of different concentrations of **Bt** and *trans*-2-dodecenal for 72 h. The MTT assay was used as a relative measure of cell viability. The obtained data are presented in Figure 3 and Table 3.

**Bt** showed remarkable cytotoxic activity, with IC_50_ values ranging from 7.93 µg/mL for A375 cells to 14.41 µg/mL for the CaCo2 cell line. An intermediate IC_50_ value for MDA-MB 231 (10.46 µg/mL) was also reported. Although the cytotoxic activity of **Bt** is significantly relevant to tumor cell lines, its selectivity index (SI: < 3) is not very high (Table 3). In fact, the IC_50_ value of non-tumor embryonic kidney cells HEK293 (10.43 µg/mL) is close to that of tumor cell lines. **Bt** was found to be particularly rich in monoterpene aldehydes, of which *trans*-2-dodecenal was the predominant component, representing 67.49% of the total **Bt** composition. Although the anti-microbial activity of *trans*-2-dodecenal is known [25,26], no data on the cytotoxic activity of this compound have been reported. *trans*-2-Dodecenal, purified from **Bt**, was analyzed for its cytotoxic activity against tumor cell lines and its involvement in the biological activity of **Bt**. The results reported in Table 3 show high cytotoxicity against both tumor and non-tumor cell lines. The IC_50_ values of the compound were lower than those of **Bt**, ranging from 1.88 µg/mL for HEK293 cells to 5.29 µg/mL for A375 cells. Even in this case, the SI was not significant (<3). Cisplatin, a conventional chemotherapy drug, was used as a positive control. The IC_50_ values of cisplatin against tumor and non-tumor cell lines are listed in Table 3. They ranged from 0.32 µg/mL for HEK293 cells to 3.45 µg/mL for MDA-MB-231. In a molar comparison of the IC_50_ values of cisplatin with those of *trans*-2-dodecenal, the latter was less cytotoxic. Notably, cisplatin as **Bt** and *trans*-2-dodecenal had an SI that was not significant (<3) for the three tumor cell lines.

**Figure 4:**
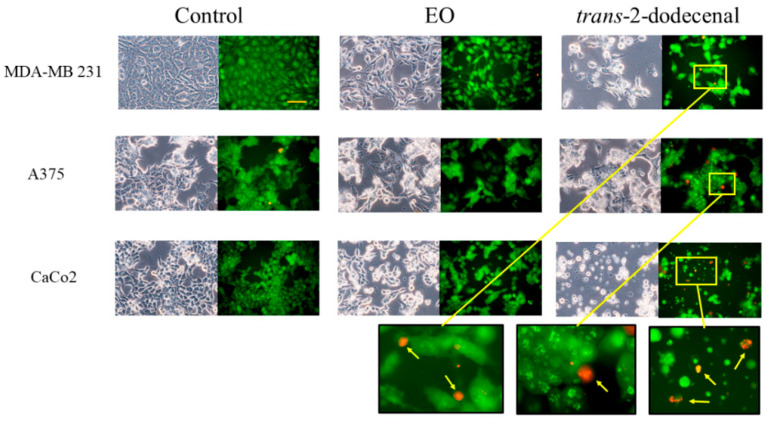
Morphological changes consistent with apoptosis were observed in MDA-MB 231, A375, and CaCo2 cells after 24 h of exposure to **Bt** and *trans*-2-dodecenal at 2 × IC_50_ concentrations. Cells were stained with acridine orange/ethidium bromide and observed using phase-contrast and fluorescence microscopy. Arrows indicate apoptotic bodies; yellow boxes indicate magnified regions. Scale bar: 50 µm.

To characterize the mechanism of action of **Bt** and *trans*-2-dodecenal on tumor cell lines, the cells were incubated in the presence of **Bt** and *trans*-2-dodecenal at the 2 × IC_50_ concentration for 2 and 24 h and then subjected to acridine orange-ethidium bromide (AO/EB) fluorescent staining. Viable cells exclude ethidium bromide and are permeable to acridine orange, which reacts with DNA to yield green nuclear fluorescence. Non-viable cells showed red/orange fluorescence because of the entry of ethidium bromide, which reacts with DNA [27]. After 2 h of incubation of the cells in the presence of **Bt** and *trans*-2-dodecenal, no morphological modifications occurred, and the green fluorescence of the cells excluded the necrotic action of the samples. Figure 4 shows the morphological changes in cells after 24 h of incubation, as observed by light microscopy. The images revealed that **Bt** and *trans*-2-dodecenal caused clear morphological changes, including cell detachment and shrinkage, as well as a reduction in cellular density compared to untreated cells. Characteristic nuclear features with chromatin condensation, which reached a late stage with dense orange areas and apoptotic bodies, were observed in AO/EB fluorescence staining, mainly in cells treated with *trans*-2-dodecenal. The effect on **Bt** was less evident, with morphological changes in cell shape and reduced presence of dense orange areas, probably due to the different concentrations of *trans*-2-dodecenal in **Bt**. These data suggest that trans-2-dodecenal acts on tumor cell lines through an apoptotic mechanism.

**Figure 5:**
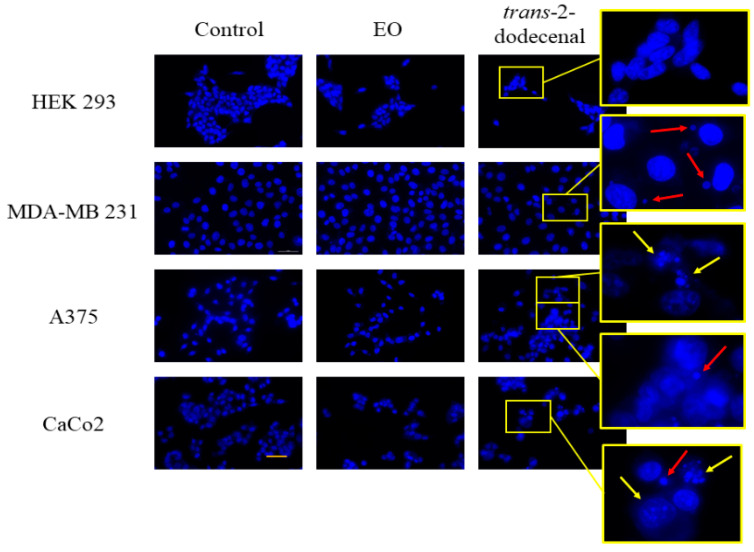
Nuclear changes induced by *B. testiculata* essential oil and *trans*-2-dodecenal in MDA-MB 231, A375, and CaCo2 cells following 24 h of treatment at 2 × IC_50_. Chromatin condensation and fragmentation were visualized using Hoechst 33258 staining under a DAPI filter on an Olympus inverted fluorescence microscope. Yellow arrows indicate apoptotic bodies; red arrows indicate micronuclei. Scale bar: 50 µm.

The treated cells were stained with fluorescent Hoechst 33258, which binds to DNA, showing the morphology and chromatin structure of the cell nuclei to further investigate the occurrence of chromatin condensation (Figure 5).

**Figure 6:**
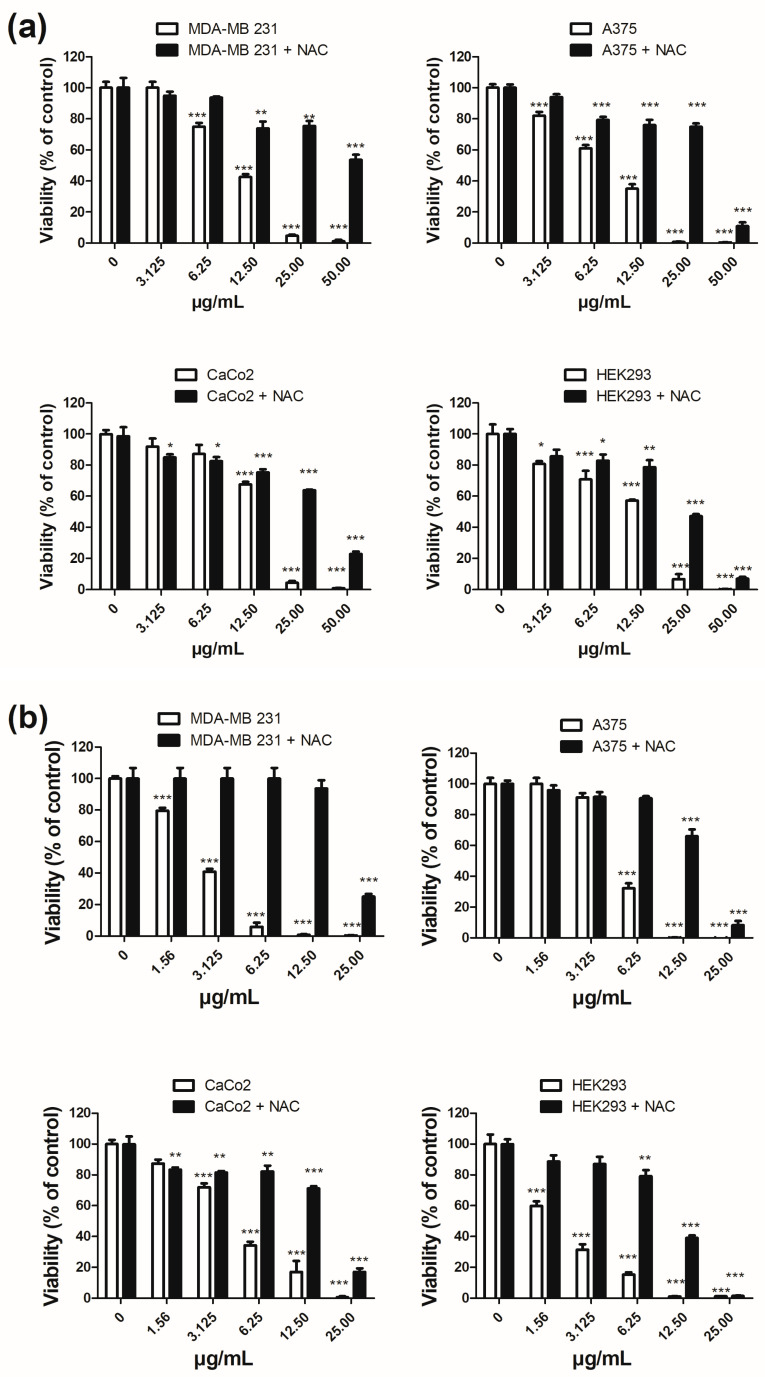
Evaluation of oxidative stress involvement in the cytotoxic effects of **Bt** (**a**) and *trans*-2-dodecenal (**b**). Cell viability was assessed in MDA-MB 231, A375, CaCo2, and HEK293 cell lines using the MTT assay after 72 h of treatment with increasing concentrations of essential oil and *trans*-2-dodecenal, in the presence (■) or absence (**□**) of 5 mM *N*-acetyl-L-cysteine (NAC). The results are expressed as the mean ± SE of three independent experiments. * *p* < 0.05, ** *p* < 0.01, *** *p* < 0.001 (vs. vehicle).

As shown in Figure 5, treatments with **Bt** had less effect on nuclear modification than treatments with *trans*-2-dodecenal. Chromatin and nuclear shapes appeared substantially ineffective in comparison with the untreated cells. In contrast, nuclei of tumor cells treated with *trans*-2-dodecenal showed chromatin condensation with the presence of apoptotic bodies in A375 and CaCo2 cell lines. In MDA-MB 231 and A375 cells, Hoechst staining revealed the presence of micronuclei in the cytoplasm of the cells, indicating exposure to a toxic substance. Micronuclei formation is associated with chromosomal instability after genotoxic stress [28]. *trans*-2-Dodecenal possesses an *α*,*β*-unsaturated aldehyde structure, and thus, can potentially act as an alkylating agent on proteins and nucleic acids [29]. Aldehydes have been reported to have cytotoxic, mutagenic, genotoxic, and carcinogenic effects [30]. Aldehydes also react with proteins, disrupting their biological activity and causing inactivation. *α*,*β*-Unsaturated aldehydes target the thiol (cysteine), imidazole (histidine), and *ε*-amino (lysine) groups of proteins to form Michael addition-type or Schiff base adducts [31]. The depletion of intracellular glutathione and oxidation of thioredoxin 1 partially account for the DNA damage-independent cytotoxicity of aldehydes [32]. These effects lead to the formation of reactive oxygen species (ROS), advanced lipoxidation end-products, and advanced glycation end-products [33]. Oxidative stress induced by **Bt** and *trans*-2-dodecenal was assessed in MDA-MB 231, A375, CaCo2, and HEK293 cells by incubating the cells in the presence and absence of *N*-acetyl-L-cysteine (NAC). The antioxidant compound was used to investigate the possible involvement of ROS in the cytotoxic effects triggered by **Bt** and its main component. As shown in Figure 6, NAC was able to reverse the cytotoxic effects of **Bt** and *trans*-2-dodecenal, even at concentrations that caused a reduction in cellular viability close to 0%. These data suggest that the cytotoxic activity of **Bt** on cell lines can be attributed mainly to the *α*,*β*-unsaturated structure of *trans*-2-dodecenal, which triggers oxidative stress. However, it cannot be excluded that other aldehydes/aliphatic acid compounds present in **Bt** might contribute to the final cytotoxic activity observed.

Although the cytotoxic activity of **Bt** is significantly relevant to tumor cell lines, its specificity is not very high. In fact, its cytotoxic activity on the normal human embryonic kidney cell line HEK293 was higher than that on tumor cell lines. However, this finding requires further investigation. HEK293 cells are immortalized cells [34] that may not correspond to the characteristics of a non-tumor cell line. Thus, it would be useful to extend the study to other tissue-specific non-tumor cell lines, such as MCF-10A (Human Mammary epithelial cells), NHEM (Normal Human Epidermal Melanocytes), and FHC (Fetal Human Colon). The mechanisms underlying the cytotoxic activity need to be examined in more detail to consider **Bt** and *trans*-2-dodecenal as potential chemotherapeutic agents. Although *α*,*β*-unsaturated aldehydes, such as citral and acrolein, are toxic [29], some long-chain *α*,*β*-unsaturated aldehydes, such as 4-hydroxynonenal, may play a regulatory role in oxidative stress [35]. Thus, it would be interesting to evaluate the role of **Bt** and its main components in cellular redox regulation.

## 3. Materials and Methods

### 3.1. Plant Materials

The aerial parts (flowers, leaves, fruits, and stems) of *B. testiculata* were collected in the area called Serre di Villalba, in the province of Caltanissetta, Sicily, Italy (37°38′19″ N 13°50′42″ E), about 830 m a.s.l., in May 2023. One of the samples, identified by Prof. Vincenzo Ilardi, was stored in the Herbarium Mediterraneum Panormitanum (PAL, voucher no. 109790) of the Botanical Garden of the University of Palermo, Italy.

### 3.2. Isolation of EO

Aerial parts (300 g) were subjected to hydrodistillation using distilled water for 3 h, according to the standard procedure described in the European Pharmacopoeia [36]. Sample yielded 0.12% of **Bt**.

### 3.3. GC-MS Analysis

The analysis of **Bt** was performed according to the procedure reported by Vaglica et al. [37]. GC-MS analysis was performed using an Agilent 7000C GC system fitted with a fused silica Agilent DB-5 MS capillary column (30 m × 0.25 mm i.d.; 0.25 μm film thickness), coupled to an Agilent quadrupole Mass Selective Detector MSD 5973 (ionization voltage 70 eV; electron multiplier energy 2000 V; transfer line temperature, 295 °C; Solvent Delay: 4 min). The oven program was as follows: temperature was increased to 40 °C for 5 min, at a rate of 2 °C/min up to 260 °C, and then isothermal for 20 min. Helium was used as the carrier gas (1 mL/min). The injector and detector temperatures were set at 250 °C and 290 °C, respectively. The EO solution (1 μL) (3% EO/hexane *v*/*v*) was injected in splitless mode; MS range 40–600.

The identification of peaks was carried out by comparison with their mass spectra and relative retention indices using the WILEY275, NIST 17, ADAMS, and FFNSC2 libraries. Kovats indices (KI) were determined using the retention times of *n*-alkanes (C_8_–C_40_) (49452-U, Supelco, Sigma-Aldrich, Milan, Italy).

### 3.4. Cell Culture

MDA-MB 231 (human breast adenocarcinoma cell line; American Type Culture Collection, ATCC, Manassas, VA, USA) and A375 (human malignant melanoma cell line; Istituto Zooprofilattico Sperimentale della Lombardia e dell’Emilia Romina, IZSLER, Brescia, Italy) were cultured in Dulbecco’s Modified Eagle’s Medium (DMEM) with 2 mM L-glutamine, 100 IU/mL penicillin, 100 µg/mL streptomycin (Corning, Manassas, VA, USA), and supplemented with 10% heat-inactivated fetal bovine serum (HI-FBS; Corning, Manassas, VA, USA). CaCo2 (human colon adenocarcinoma cell line; American Type Culture Collection, Manassas, VA, USA) was cultured in DMEM with 2 mM L-glutamine, 100 IU/mL penicillin, 100 μg/mL streptomycin, 1% Non-Essential Amino Acid (NEAA), and supplemented with 10% HI-FBS. HEK293 (human embryonic kidney cell line; kindly donated by Dr. Michela Buccioni, School of Pharmacy, University of Camerino, Camerino, Italy) was cultured in Eagle’s Minimum Essential Medium (MEM) with 2 mM L-glutamine, 100 IU/mL penicillin, 100 µg/mL streptomycin, and supplemented with 10% HI-FBS. The cells were maintained in an incubator at 37 °C in a humid atmosphere enriched with 5% CO_2_ and 95% relative humidity.

### 3.5. MTT Assay

The cytotoxic activity of **Bt** and its main component (*trans*-2-dodecenal, Merck Life Science S.r.l., Milano, Italy) was determined using the 3-(4,5-dimethylthiazol-2-yl)-2,5-diphenyltetrazolium bromide (MTT; Sigma-Aldrich, St. Louis, MO, USA) method [38,39]. The reduction of tetrazolium salt to formazan by mitochondrial succinate dehydrogenase was used to evaluate the viability of cancer cells, as previously described [40]. MDA-MB 231, A375, CaCo2, and HEK293 cells (2 *×* 10^4^ cells/mL) were added to 96-well plates, incubated for 24 h, and exposed to different concentrations of **Bt** (0.78–200 µg/mL; vehicle: ethanol) and *trans*-2-dodecenal (0.39–200 µg/mL; vehicle: ethanol) for 72 h in a humidified atmosphere of 5% CO_2_ at 37 °C. At the end of the incubation period, each well received 10 µL of MTT (5 mg/mL in phosphate-buffered saline, PBS), and the plates were incubated for 4 h at 37 °C. The supernatant was then removed, and 100 µL of dimethyl sulfoxide (DMSO; Sigma-Aldrich) was added to dissolve the formazan crystals. Absorbance was measured using a microplate reader (FLUOstar Omega, BMG Labtech, Milan, Italy) at 540 nm. The percentage of viability was calculated using the following equation:

$$Viability\;\%=\;\frac{A_{sample}-\;A_{blank}}{A_{vehicle}-A_{blank}}\;\times 100\%$$

*A* = Absorbance at 540 nm.

In a parallel experiment, **Bt** and its main component were incubated in the presence of 5 mM NAC (N-acetyl-L-cysteine; Sigma-Aldrich). The anti-cancer drug cisplatin (0.01–50 μg/mL) was used as a positive control. The experiments were conducted in triplicate. The 50% cytotoxic concentration (IC_50_) was defined as the compound concentration required to reduce cell viability by half. The IC_50_ values were determined using GraphPad Prism 5 (GraphPad Software, San Diego, CA, USA). IC_50_ values were obtained from graphs plotted using a non-linear regression equation log (inhibitor) versus response—variable slope.

The selectivity index (SI) was calculated as the ratio of the IC_50_ value for HEK293 cells divided by the IC_50_ values for A375, MDA-MB 231, or CaCo2 cells. The SI value indicates the degree of selective toxicity of a given **Bt** or its main components to the cancerous cell lines tested, relative to that of the non-tumor cell line. Any sample of an SI value > 3 was considered to indicate high selectivity [24].

### 3.6. Acridine Orange and Ethidium Bromide Staining

Characteristic apoptotic or necrotic morphological changes were investigated by fluorescent microscopy using acridine orange and ethidium bromide staining. MDA-MB 231, A375, and CaCo2 cells were seeded at a density of 2.5 × 10^5^ cells/mL. After 24 h, the cells were treated with 2 × IC_50_ values of **Bt** and *trans*-2-dodecenal for 2 and 24 h. After incubation, the cells were washed with PBS and mixed with 20 μL of a fluorochrome mixture (50 μg/mL acridine orange and 50 μg/mL ethidium bromide in PBS) in 500 µL of PBS for 3 min. The samples were examined under an Olympus IX71 fluorescent inverted microscope with an appropriate combination of filters and recorded using an Olympus DP90 digital camera. Viable cells had intact, bright green nuclei. Early apoptotic cells contained a bright green nucleus with condensed chromatin, whereas late apoptotic cells contained a red/orange nucleus showing chromatin condensation [41].

### 3.7. Hoechst Nuclear Staining

MDA-MB 231, A375, CaCo2, and HEK293 cells (1 × 10^6^ cells/mL) were exposed to **Bt** and its main component for 24 h at a concentration corresponding to 2x IC_50_ values and then fixed with methanol/acetic acid (3:1). Samples of fixed cells were incubated with Hoechst 33258 nuclear stain (0.05 µg/mL in PBS) (Sigma-Aldrich, St. Louis, MO, USA) for 10 min. The stain was removed, and the coverslip was mounted in a drop of buffered glycerol mountant (0.4 M Na_2_HPO_4_, 0.2 M citric acid, and 50% glycerol). Nuclei were observed using an Olympus IX71 (Olympus Italy s.r.l., via San Bovio 1, Segrate, MI, Italy) inverted fluorescence microscope and recorded using an Olympus DP90 digital camera.

### 3.8. Statistical Analysis

Statistical analyses were performed using one-way ANOVA, followed by Tukey’s post-hoc test to test for statistical differences between groups. Significance was represented by * *p* < 0.05, ** *p* < 0.01, *** *p* < 0.001. The statistical analysis software used was GraphPad InStat v3.0 (GraphPad Software, San Diego, CA, USA).

## 4. Conclusions

This study provides new insights into the chemical composition of *Bifora testiculata* essential oil, confirming its aldehyde-rich nature and highlighting its potential as a source of bioactive compounds. The predominant presence of *trans*-2-dodecenal (67.49%) distinguished the Sicilian accession and aligned with previous findings from a Greek population, reinforcing the idea that aldehydes are key chemotaxonomic markers within the *Bifora* genus. However, notable differences in minor constituents suggest potential chemotype variability among different accessions. Given the known bioactive properties of *trans*-2-dodecenal and structurally related aldehydes, the essential oil and its main component exhibit remarkable cytotoxic activity against tumor and non-tumor cell lines. Preliminary data suggest that **Bt** and its main compound, *trans*-2-dodecenal, act on cancer cell lines, triggering apoptosis through ROS stimulation. However, more research is needed to demonstrate the safety of essential oils and *trans*-2-dodecenal in non-tumor cells, as well as to elucidate their role in cellular redox regulation. In light of these results, the elucidation of the molecular mode of action of **Bt** and *trans*-2-dodecenal and the pathways involved in the apoptotic mechanism will be investigated in future works. In addition, the study will be extended to other tumor and non-tumor cell lines to better clarify their anti-tumor potential.

## Figures and Tables

**Table 1:** Chemical composition (%) of *B. testiculata* essential oil collected in Sicily, Italy, as determined by GC-MS.

No.	Compounds ^a^	KI ^b^	KI ^c^	Area (%) ^d^
1	*trans*-2-Hexenal	852	855	0.13
2	Octanal	1002	1006	0.06
3	*p*-Cymene	1020	1022	0.01
4	Sylvestrene	1025	1028	0.01
5	(*E*)-*β*-Ocimene	1048	1050	0.02
6	*trans*-3-Undecene	1086	1088	0.04
7	Nonanal	1102	1102	0.07
8	*cis*-4-Decenal	1192	1193	0.03
9	*trans*-4-Decenal	1195	1197	0.08
10	Decanal	1205	1207	1.95
11	*cis*-2-Decenal	1253	1255	3.25
12	*trans*-2-Decenal	1257	1263	0.17
13	2-Octylfuran	1280	1284	0.11
14	*n*-Tridecane	1295	1300	0.14
15	Undecanal	1303	1310	0.14
16	*cis*-2-Undecenal	1347	1350	0.03
17	*trans*-2-Undecenal	1360	1361	0.42
18	*trans*-4-Undecenal	1396	-	3.38
19	Dodecanal	1408	1412	4.50
20	*cis*-2-Dodecenal	1460	1464	2.91
21	*trans*-2-Dodecenal	1465	1472	67.49
22	*trans*-2-Tridecenal	1550	1553	0.79
23	Dodecanoic acid	1564	1567	0.83
24	13-Tetradecenal	1602	1608	0.32
25	Tetradecanal	1604	1613	0.38
26	*trans*-2-Dodecenoic acid	1631	-	4.55
	Monoterpene hydrocarbons			0.04
	Hydrocarbons			0.18
	Aldehydes			86.10
	Aliphatic acids			5.38
	Other			0.11
	Total			91.81

^a^ Components listed in order of elution on an DB-5 MS apolar column; ^b^ Kovats indices on a DB-5 MS; ^c^ Kovats indices based on literature (https://webbook.nist.gov; accessed on 10 June 2025); ^d^ Relative percentage values of the separated compounds calculated from integration of the peaks.

**Table 3:** In vitro cytotoxic activity of *B. testiculata* essential oil and *trans*-2-dodecenal on human cancer and non-cancer cell lines.

	Cell line (IC_50_ µg/mL) ^a^			
	MDA-MB 231 ^b^	A375 ^c^	CaCo2 ^d^	HEK293 ^e^
EO	10.46	7.93	14.41	10.43
95% C.I. ^f^	9.75–11.22	7.36–8.56	13.41–15.48	8.72–12.48
SI ^g^	0.99	1.31	0.72	
*trans*-2-dodecenal	2.66 (14.59) ***	5.29 (29.02) ***	4.74 (26.00) ***	1.88 (10.31) ***
95% C.I.	2.56–2.76	5.02–5.57	4.36–5.16	1.69–2.08
SI	0.71	0.36	0.40	
Positive control				
Cisplatin	3.45 (11.50) ***	0.55 (1.83) ***	3.28 (10.93) ***	0.32 (1.07) ***
95% C.I.	2.73–3.64	0.49–0.63	2.33–3.75	0.29–0.36
SI	0.09	0.58	0.097	

^a^ IC_50_ = The concentration of compound that affords a 50% reduction in cell growth (after 72 h of incubation). ^b^ Human breast adenocarcinoma cell line. ^c^ Human malignant melanoma cell line. ^d^ Human colon adenocarcinoma cell line. ^e^ Human embryonic kidney cell line. ^f^ Confidence interval. ^g^ Selectivity index, calculated as the ratio of IC_50_ for HEK293 cells/tumor cell lines; an SI value > 3 indicates high selectivity [24]. Values in brackets are in µM. ND: not determined. *** *p* < 0.001, compared with EO evaluated using the Tukey multiple comparison.

## Data Availability

The original contributions presented in this study are included in the article. Further inquiries can be directed to the corresponding author.
